# Capturing the Multiscale Nature of Bone Behavior: Classical, Data-Driven and Hybrid Techniques

**DOI:** 10.1007/s10439-026-04043-7

**Published:** 2026-03-02

**Authors:** Melika Mohammadkhah, Ardeshir Savari, Sandra Klinge

**Affiliations:** 1 Chair of Structural Mechanics and Analysis, Institute of Mechanics, Technische Universität Berlin, Straße des 17. Juni 135, 10623 Berlin, Germany; 2 Department of IT and Engineering, IU International University of Applied Sciences-Campus Berlin, Frankfurter Allee 71-77, 10247 Berlin, Germany; 3 Department of Mechanical Engineering, Petroleum University of Technology, Ayatollah Behbahani Expy, 6MG3V34 Ahvaz, Iran

**Keywords:** Bone remodeling, Bone biomechanics, Classical models, Data-driven models, Neural networks, Finite element multiscale modeling

## Abstract

**Abstract:**

The complexities of bone architecture, with its hierarchical organization and varying spatiotemporal scales, necessitate advanced modeling techniques to capture its mechanical behavior precisely. This review aims to highlight recent trends in capturing the multiscale nature of bone using two primary computational approaches: classical and data-driven frameworks. Each class is assessed regarding its versatility in achieving scale dimensions, modeling complex behavior, integrating biological data, and balancing computational efficiency and interpretability. In addition, hybrid techniques have been shown to offer future avenues for promising robust and generalizable modeling. Therefore, particular attention has been given to the synergy between these techniques. A hierarchical decision matrix is proposed to translate this review into actionable guidance, shedding light on the selection or combination of appropriate techniques based on specific application contexts, such as data availability, modeling objectives, and computational constraints. This review aims to serve as both a state-of-the-art synthesis and a practical reference for future advancements in multiscale bone biomechanics.

**Graphical Abstract:**

**Figure:**
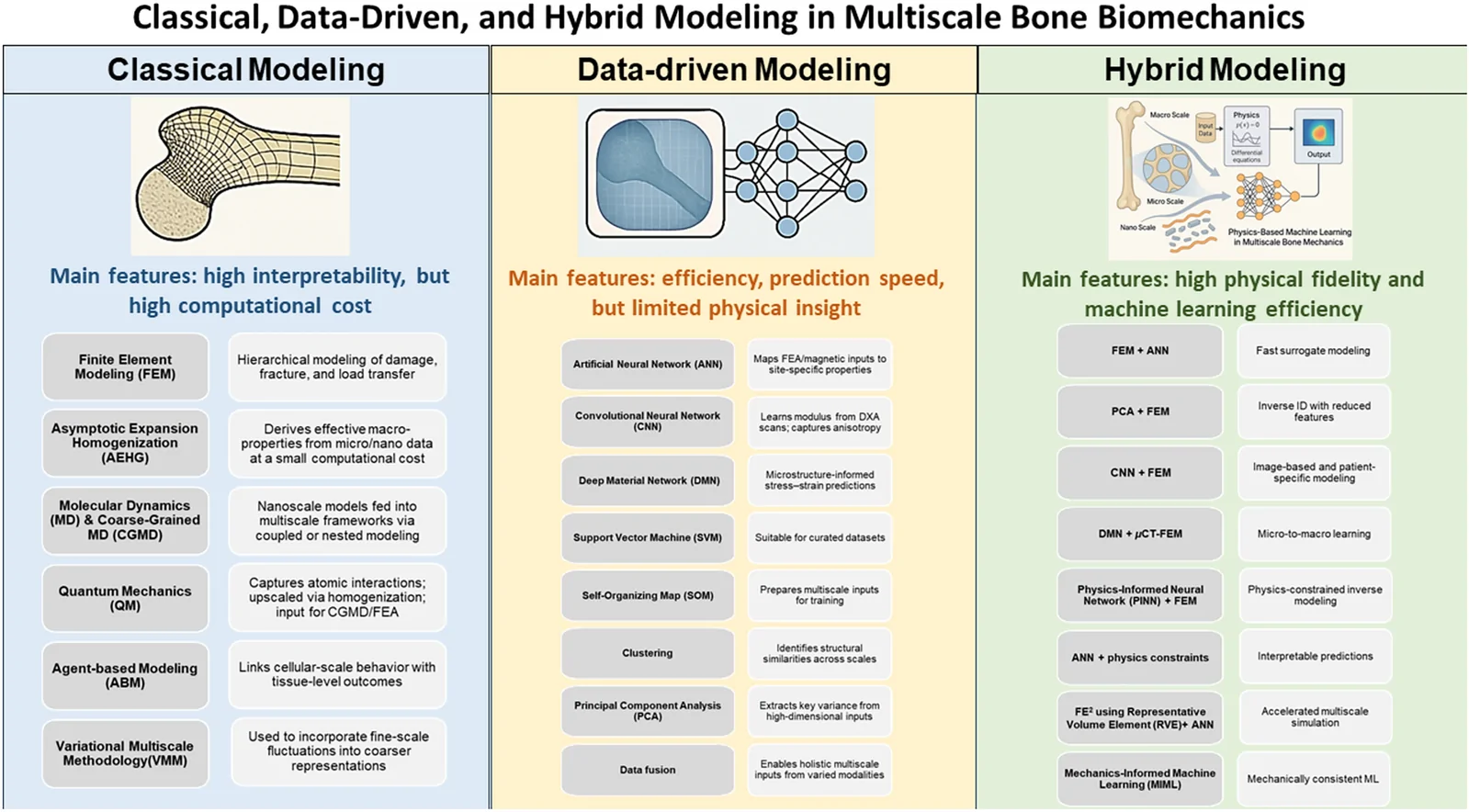

## Introduction

Bone mechanics plays an essential role in addressing the global health and economic burden associated with bone fragility and fractures [1]. Diseases such as osteoporosis affect approximately 200 million people worldwide and contribute to an estimated 8.9 million fractures annually [1–5], resulting in severe disability and mortality, with one in three patients dying within a year of fracture. These conditions collectively impose more than $35 billion in costs on the global healthcare system [6]. While research into bone health is extensive, a comprehensive understanding of bone's mechanical behavior remains elusive. A major challenge lies in elucidating how macroscopic deformations translate to nanoscale processes within the hierarchical bone structure. Therefore, advancing our understanding of bone’s complex mechanical behavior is essential, as it holds the potential to open new paths for diagnostics and therapeutic strategies in advanced clinical care [1].

Bone's exceptional strength and toughness arise from its hierarchical multiscale architecture, which spans the molecular interactions through fibrillar and extracellular levels to osteonal and organ scales. This hierarchy motivates multiscale modeling as a central strategy for understanding both healthy and diseased bone. Evaluating bone health using traditional clinical indicators such as bone mineral density (BMD) provide useful information on bone fracture risk and is currently used by densitometrists [7, 8]. However, such indicators fail to capture critical aspects of bone quality related to nanoscale and microscale mechanisms. Emerging evidence has shown that nanoscale tissue interactions, such as collagen malformation in osteogenesis imperfecta or impaired osteogenesis in diabetes, play a crucial role in determining bone behavior [9]. Recently advanced experimental tools such as high-resolution imaging techniques, e.g., synchrotron imaging, micro-computed tomography (micro-CT), together with computational techniques, e.g., molecular dynamics (MD) and finite element method (FEM) and non-FEM approaches, have made it possible to investigate bone behavior at increasingly fine scales. These developments have significantly advanced our ability to assess mineral quality and nanoscale interactions, which are now recognized as key determinants of bone strength.

Studies have demonstrated that at the sub-micron/nanoscale (below 50 µm), bone does not fail by crack formation but rather through elasto-plastic deformation mechanisms [10, 11], including ductile sliding between hydrated mineral crystals and subsequent rupture of collagen crosslinks, as established by non-FEM hierarchical micromechanics [12] and corroborated in recent hierarchical elastoplastic formulations [11]. Despite this well-established physical understanding, embedding these nanoscale mechanisms into efficient predictive computational frameworks remains less investigated and the mechanisms responsible for arresting damage are still not fully understood [1]. In addition to fracture mechanics, bone remodeling further demands sophisticated computational approaches to unravel its complex multiscale and multiphysics nature [13, 14]. Traditional tools often fall short in resolving the intricate stress-strain transfer mechanisms across scales.

**Fig. 1:**
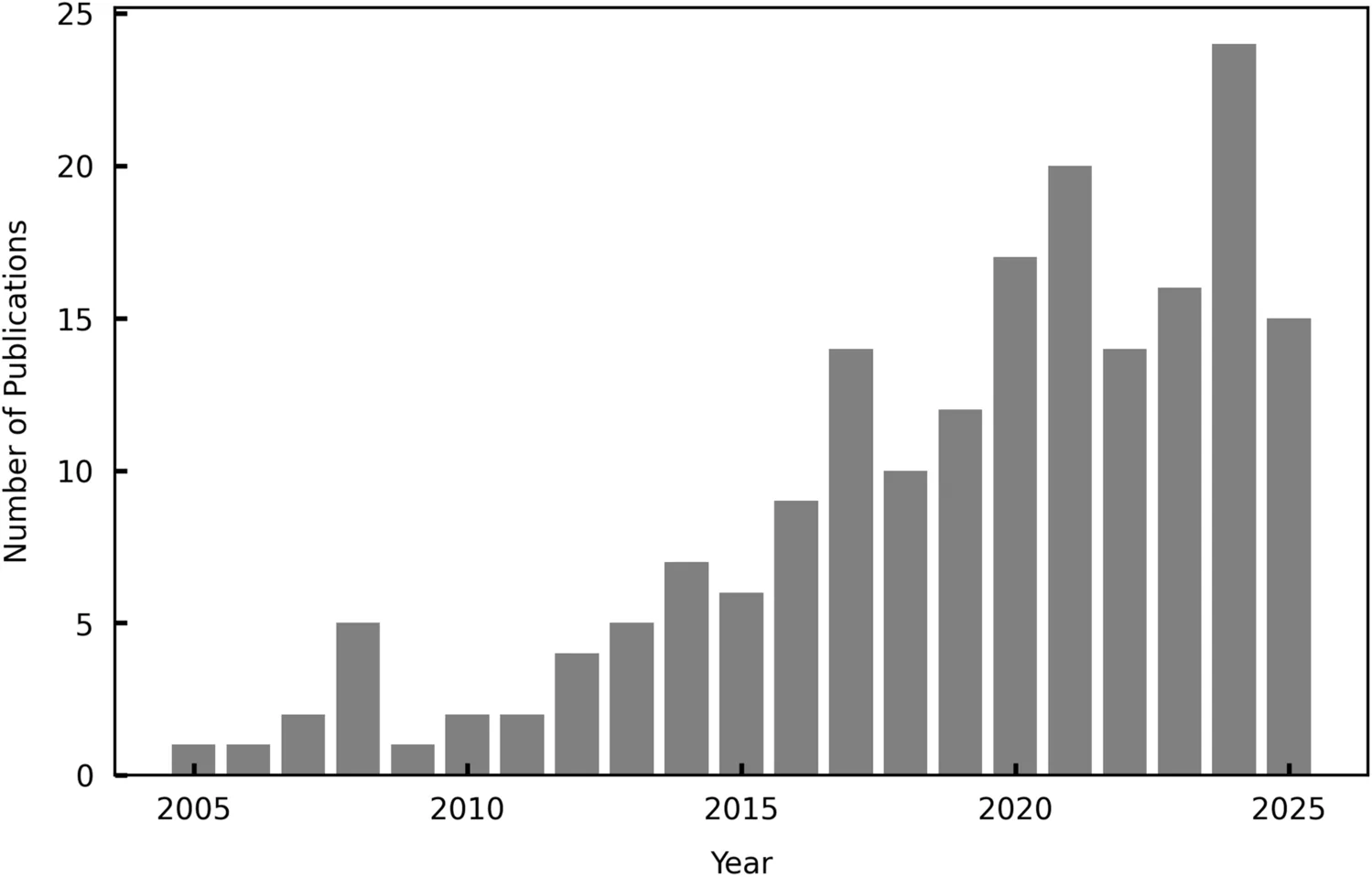
Number of published studies reviewed in this work on multiscale bone biomechanics over the past two decades

Traditional experimental approaches in bone research are invaluable but expensive, time-consuming, and ethically challenging, especially when collecting human tissue samples for subsequent analysis. However, computational modeling is a powerful extension to experimental investigations, providing a cost-effective alternative to explore the complex mechanical behavior of bone across multiple scales without the ethical and practical constraints of experimental sampling [1, 15–23]. Among computational approaches, classical techniques offer science-based mechanistic understanding, and are limited to fully capture nonlinear behavior [1, 24, 25], but data-driven techniques are able to model complex, nonlinear dependencies across large datasets obtained from experiments and high-fidelity simulations [26–28]. However, purely data-driven approaches may lack physical interpretability and become unreliable when data are sparse or heavily corrupted by noise. Such considerations have fueled the growing interest in hybrid modeling strategies that synergize classical approaches and data-driven paradigms [29, 30]. A quantitative assessment of the literature reviewed in this study (Fig. 1) reveals a steady increase in yearly publications over the past two decades, reflecting high interest in unraveling bone’s multiscale behavior and the parallel emergence of classical, data driven, and hybrid computational approaches. This steady growth underscores the expanding relevance of multiscale modeling and motivates the need for an integrated and systematic synthesis such as the one provided here.

Therefore, this review places primary emphasis on studies published within the last decade, where the field has seen the most substantial growth and methodological development and systematically examines recent advances in computational modeling techniques that integrate data-driven approaches with existing physics-based theoretical frameworks to capture bone's multiscale hierarchical behavior. By bridging these domains, the review aims to accelerate our understanding of bone mechanics across scales, ultimately enhancing the prediction of bone behavior and supporting the development of improved diagnostic tools and treatment strategies for bone diseases. This review stands apart from previously published articles by offering the first integrated synthesis of three major paradigms in multiscale bone biomechanics: classical physics-based models (particularly FEM), data-driven machine learning (ML) frameworks, and emerging hybrid physics-informed methods, and mechanics-informed ML. None of prior reviews have comprehensively examined how these methodologies complement each other across hierarchical bone scales. Furthermore, this work introduces a decision matrix that allows researchers and clinicians to select the most appropriate modeling approach based on scale, data availability, interpretability demands, and computational constraints. The authors believe that this review provides a holistic and forward-looking framework for advancing multiscale bone modeling by unifying methodological advances and discussing their validation, and translational potential.

The review begins by outlining the multiscale nature of bone mechanics in "Multiscale Nature of Bone" section, followed by an exploration of classical approaches especially FEM and relevant techniques that incorporate established biomechanical principles ("Classical Multiscale Techniques" section). In "Data-Driven Multiscale Techniques" section, data-driven frameworks that leverage experimental or simulation datasets, often without explicitly encoding physical laws are investigated. Building on this, we examine emerging hybrid approaches in "Hybrid Multiscale Techniques" section, with a focus on physics-informed ML techniques that integrate physical constraints into data-driven models, effectively bridging empirical knowledge and computational intelligence. Special emphasis is placed on the verification and validation of these, assessing their reliability and applicability in both clinical and biomechanics-focused contexts. The review also emphasizes the practical applications of these computational approaches in bone research in "Potential Applications of Classical, Data-Driven and Hybrid Models" section. It compares insights across different modeling paradigms, discussing their respective advantages and limitations, and highlights key challenges ("Discussion" section). Finally, Sect. 8 concludes the review by outlining the future scope of the topic and promising directions for further research.

## Nomenclature

**Table:** 

Abbreviations	Description	Abbreviations	Description
CGMD	Coarse-Grained Molecular Dynamics	PCA	Principal Component Analysis
CT	Computed Tomography	PINN	Physics-Informed Neural Network
DXA	Dual-Energy X-ray Absorptiometry	PINO	Physics-Informed Neural Operator
HOMS-PINN	Higher-Order Multiscale PINN	QCT	Quantitative Computed Tomography
HR-pQCT	High-Resolution Peripheral QCT	QM	Quantum Mechanics
LCN	Lacuno-Canalicular Network	RVE	Representative Volume Element
MCF	Mineralized Collagen Fibril	SOM	Self-Organizing Map
MCM	Mineralized Collagen Microfibril	SVM	Support Vector Machine
MD	Molecular Dynamics	TCPINN	Theory-Constrained PINN
MIML	Mechanics-Informed Machine Learning	VMM	Variational Multiscale Methodology
NNRPIM	Natural-Neighbor Radial-Point Interpolation	NCP	Noncollagenous Protein
PBML	Physics-Based Machine Learning		

## Multiscale Nature of Bone

**Fig. 2:**
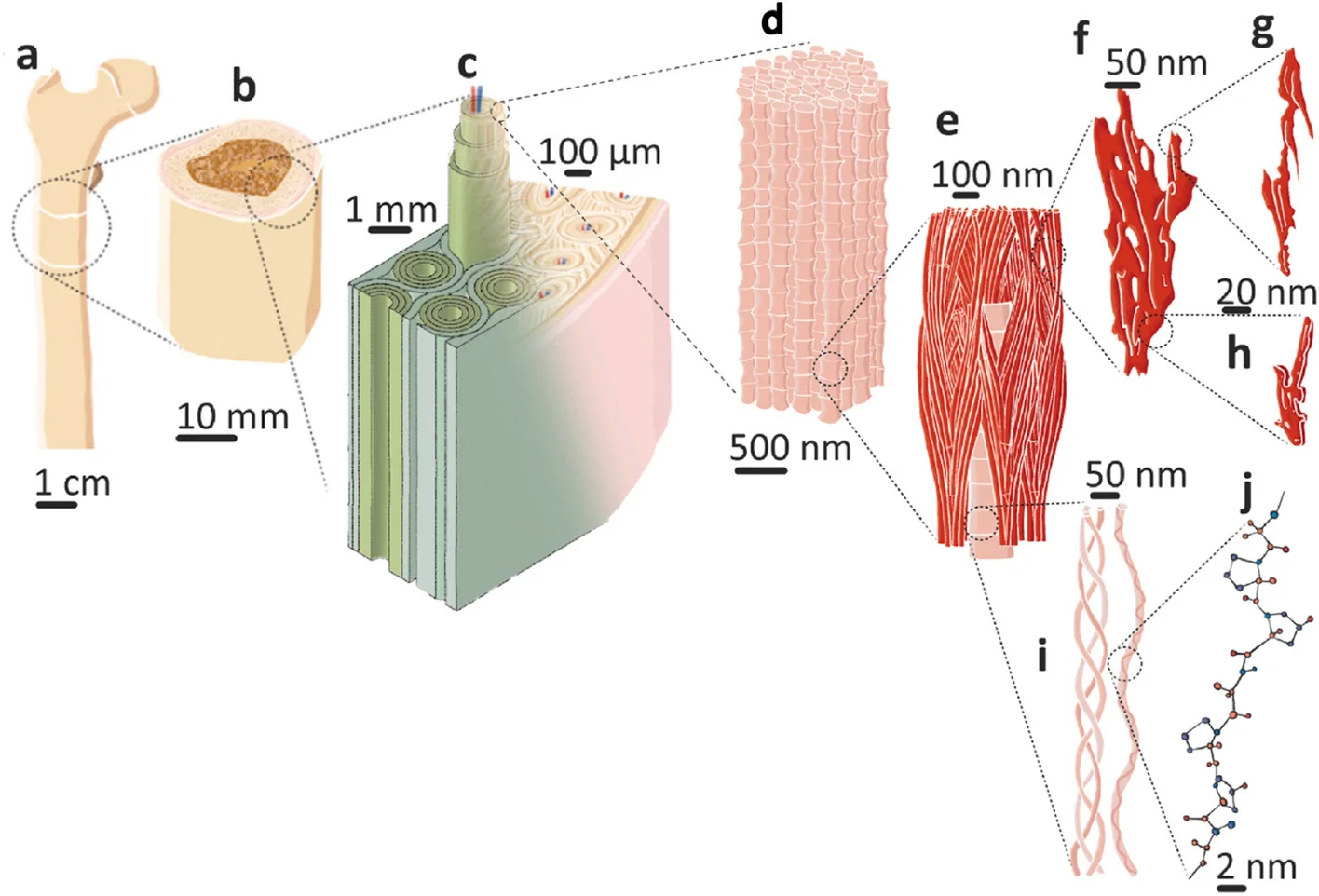
Levels of hierarchy constructed in bones. **a** Femur, **b** cross-section of the femur showing the fibrolamellar structure which consists of outer fibrolamellar, middle cortical bone, and inner marrow cavity, **c** close differentiation of the Haversian System, built of collagen fibers and mineral crystals (right), compared to the bioinspired composite built by carbon and glass fibers with epoxy resin (left), **d** collagen fibers depicted as bundles of collagen fibrils, **e** mineralized collagen fibrils, **f** mineral aggregates, including hydroxyapatite in forms of **g** platelet and **h** acicular crystals, **i** molecular structure of collagen fibers (tropocollagen), and **j** close-up view of a polypeptide chain (adapted with permission from [40, 41])

The hierarchical structure of bone underpins its remarkable mechanical performance and adaptability, resulting from interactions across multiple length scales (Fig. 2). This section explores the architecture of bone from the macroscopic to the molecular level and explains how this hierarchical organization translates into mechanical behavior and corresponding biological responses.

### Macroscale (>1 mm)

At the macroscopic scale, bone tissue is classified into two primary types (Fig. 2a, b): compact cortical bone and the more porous trabecular (or cancellous) bone [31, 32]. Cortical bone, containing about 80% of the total skeletal mass, is a thick, compact layer of bone outside and around the long bone shafts, providing high stiffness and strength within load-bearing conditions [33, 34]. Trabecular bone is distinguished by its porous architecture, featuring a network of trabeculae that form marrow-filled cavities. It is typically found in the epiphyseal and metaphyseal regions of long bones, as well as in the pelvis, flat bones (e.g., ribs, skull), and vertebrae [1, 35–37]. The transition from cortical to trabecular bone is gradual rather than abrupt; an adaptation-driven design that leverages material gradients to efficiently transfer mechanical loads between distinct tissue types [38]. This macroscale organization not only enhances the bone’s capacity to protect internal organs, anchor muscles for locomotion, and support body weight, but also accommodates structural adaptations tailored to the functional demands of long, flat, and short bones [39].

Several structures such as the periosteum, endosteum, and bone marrow, are intricately connected with the primary bone tissues. The periosteum is a dense, vascularized fibrous membrane that covers the external surface of bones (except at articular ends), serving to anchor muscles and ligaments, supply nutrients, and facilitate bone growth and repair. The endosteum is a thin, delicate membrane that lines the inner surfaces of bones, and forms another bone remodeling and repair site. Bone marrow is a soft, spongy tissue found within the medullary (marrow) cavities of long bones and in the trabecular (spongy) regions of flat and irregular bones. It plays a central role in blood cell production and fat storage, depending on its type [42]. The macroscale architecture of bone is not static; it undergoes continuous structural adaptation in response to mechanical loading through remodelling—a dynamic process that converts mechanical stimuli into biochemical and cellular signals via mechanotransduction [43].

Microstructural differences in trabecular and cortical bones facilitate different mechanical and physiological functions. The trabecular bone has a modulus value of 1–2 GPa with porosity up to 90% that favors metabolic activity and marrow accommodation [44], whereas with only 3–5% of its porosity and a modulus of 16–23 GPa [45], the cortical bone provides a rigid structure. Their anisotropic behavior is due to the orientation of osteons and trabeculae in principal stress directions, optimizing resistance to mechanical load without gaining substantial weight [46, 47]. Furthermore, dynamic remodeling of trabecular bone occurs via the densification of plate-like structures in high-stress regions and a configuration of rod-like structures in low-stress areas, giving a load-adaptive architecture supporting the mechanical effectiveness of bone [48–50].

### Microscale (1–500 μm)

Bone exhibits a highly complex hierarchical structure, characterized by the deposition of mineralized collagen fibrils (MCFs) into progressively larger structural units whose mechanical properties arise from their organization (Fig. 2c) [1]. Lamellae, the fundamental building blocks, assemble into osteons in cortical bone and trabeculae in trabecular bone, enabling bone to adaptively optimize strength, stiffness, and toughness in response to diverse metabolic and mechanical demands [1]. These thin, 3–7 μm layered sheets formed by regionally oriented MCFs modulate local stress fields and mechanical adaptability. The MCFs variability enables the calibration of elastic and failure properties under tensile or compressive loads [41, 51, 52]. The mineral phase of bone, primarily composed of hydroxyapatite (HA), forms ellipsoidal aggregates that align with collagen fibrils but remain loosely attached to them [53]. These aggregates organize into tesselles—distinctive, twisted, tessellated arrangements of micron-scale mineral clusters—that follow the orientation of collagen fibers. This architecture enhances resistance to compressive loads and promotes crack deflection. Together, these features make lamellae the microscale foundation of bone’s hierarchical strength and durability [1, 54, 55]. Laminated structures of cortical bone are arranged into osteons, which are cylindrical units averaging ~ 200 μm in diameter, thus enhancing stiffness and strength in weight-bearing areas [56, 57]. The lacuna-canalicular network (LCN) formed by osteocyte cells within the lacunae communicate through the canaliculi, enabling mechanosensing and strongly influencing remodeling responses [58, 59]. In trabecular bone, lamellae form interconnected plates and rods, measuring 100–500 μm in thickness and oriented along principal stress directions, granting a high strength-to-weight ratio and energy absorption capacity to the overall structure [1, 57, 60]. While trabecular bone is less dense than cortical bone, it is metabolically much more active and, therefore, maintains mineral homeostasis well and engages in mechanical adaptability [51].

### Nanoscale (1–100 nm)

Bone has an intricate composite architecture at the nanoscale, primarily composed of Type I collagen fibrils, HA nanocrystals, noncollagenous proteins (NCPs), and water (Fig. 2d–j). Collagen molecules undergo self-assembly into fibrils with a characteristic ~ 67 nm D-periodic structure, forming gap regions that act as nucleation sites for HA crystals [58, 61]. HA constitutes ~ 60% of bone mass and mainly exists in poorly crystalline, platelet-like crystals of varied morphology and solubility under a given set of conditions, particularly concerning ionic substitutions [1, 3, 62]. NCPs, limited in content, are known to regulate mineralization, provide adhesion between collagen and minerals, and assist energy dissipation by forming sacrificial bonds [63]. Whether gratis or bound, water represents an essential player in viscoelasticity and toughness owing to facilitating fibrillar sliding and rearrangement of hydrogen bonds under load [64]. These constituents are then combined to form an MCF, the fundamental nanostructural unit governing the mechanical competence and biological functionality of bone across scales and provides key insight into the design of next-generation biomaterials [65].

## Classical Multiscale Techniques

### FEM

Classical methods depend on physical principles, with FEM being extensively employed in the field of multiscale bone mechanics. Studies on multiscale finite element (FE) modeling of cortical bone, summarized in Table 1, have effectively captured its anisotropic mechanical behavior, hierarchical failure mechanisms, and complex electromechanical interactions. Several investigations [66–68] employed nano-to-micro or micro-to-macro strategies to simulate damage initiation, stiffness degradation, and fracture evolution. These models often integrate cohesive zone modeling (CZM) at collagen–mineral interfaces [66] or utilize 3D representative volume element (RVE) homogenization based on collagen network topology. For instance, bottom-up formulations incorporating lamellar and interlamellar properties have successfully reproduced brittle failure modes characteristic of osteons [58]. Mineral phase heterogeneity has been linked to anisotropic elasticity across cortical compartments [24]. Additionally, researchers have modeled viscoelasticity and time-dependent anisotropy in cortical bone [7, 69], incorporating statistical sensitivity analyses and rate-dependent damage-coupling approaches. Recent models address flexoelectric- and piezoelectric-driven remodeling [70–74] magnetoelectric porosity sensing [15], and magneto-mechanical diagnostic markers [75], offering new insights into the link between microscale porosity and macro-level diagnostics, especially relevant in osteoporosis. Modeling efforts typically focus on anatomical regions such as the femur, vertebrae, and distal radius, emphasizing osteonal remodeling, age-related degeneration, or bone–implant interactions [76, 77]. Despite significant progress, challenges remain, including oversimplified geometry, limited incorporation of biochemical signaling and marrow interactions, and insufficient experimental validation for electromechanical and remodeling mechanisms.

**Table 1:** Multiscale FE studies: cortical bone

Multiscale focus	Year	Key contributions	Limitations	References
Dynamic macro–micro load transfer	2025	Transient macro–micro stress amplification	No soft tissue; simplified microstructure	[76]
Nano–micro interface failure	2024	Cohesive modeling of collagen–mineral interfaces	2D modeling; excludes 3D fibril networks	[66]
Collagen network homogenization	2024	3D RVE homogenization from collagen topology	Neglects rate dependence; simplified cell–matrix interaction	[67]
Microstructure-driven magnetomechanics	2024	RVE variability for magnetic diagnostics	Idealized RVEs; excludes fluid effects	[75]
Hierarchical damage mechanics	2023	Anisotropic damage onset across mineral content	Excludes nanoscale bonds; ignores porosity	[68]
Flexoelectric-induced remodeling	2023	Strain-gradient triggered multiscale remodeling	Oversimplifies BMU dynamics; lacks 3D crack closure	[70, 73, 74]
Aging effects on femoral neck mechanics	2022	Multiscale FE models (organ to osteocyte LCN) show elderly cortical bone strains/SEDs 2–14 × higher than young	Simplifies LCN geometry; ignores muscle force dynamics	[78]
Osteon elasto-damage	2022	Bottom-up modeling with lamellar interaction	Excludes cement lines; no chemo-biological coupling	[58]
Mineral distribution in lamellar bone	2022	Elasticity linked to intra/extra HA content	Assumes perfect bonding; ignores NCP variability	[24]
Magnetoelectric osteoporosis detection	2022	Coupled electric–magnetic simulation of porosity loss	Simplified RVEs; lacks validation	[15]
Viscoelastic–anisotropic coupling	2021	Time-dependent anisotropy from viscoelasticity	Excludes hydration effects; homogenized bone	[69]
MCF orientation to lamellar fracture	2020	MCF-based crack propagation with orientation	Simplifies collagen-glycation; ignores porosity	[79]
Cortical–trabecular load sharing	2018	Boundary condition refinement for strain sharing in distal radius	Partial volume artifacts; elderly bias	[77]
Two-way viscoelastic–damage coupling	2017	Damage evolution from global–local rate dependence	Simplified damage laws; no multiaxial validation	[7]
Femur fracture prediction	2017	Adaptive reduced-order homogenization for fracture risk	Limited clinical validation	[80]

Overall, Table 1 shows how multiscale FE formulations have increasingly advanced the mechanistic understanding of cortical bone by incorporating hierarchical organization, anisotropy, and coupled electromechanical processes. Despite this progress, most studies continue to rely on geometric simplifications or partial representations of biochemical and nanoscale interactions, highlighting opportunities for future model refinement.

Multiscale models of cancellous bone, as reviewed in Table 2, have primarily focused on remodeling, damage progression, and energy dissipation, attributes closely tied to its porous and highly adaptable architecture under physiological and pathological loading conditions. Nonlinear homogenization at the microscale has been linked to macro-scale mechanical responses, enabling predictions of damage evolution and optimization of implant designs [81, 82]. Some studies have modeled progressive osseointegration by comparing μCT- and CT-based geometries [81], while others have predicted viscoelastic behavior using constitutive laws calibrated through microindentation experiments [83]. Extensive multiscale simulations of trabecular structures have been conducted to investigate directional anisotropy and remodeling thresholds under dynamic or patient-specific loading, particularly in the vertebrae, femoral head, and dental implant [77, 81, 82]. Although these models offer enhanced biomechanical realism, several limitations persist: simplification of trabecular geometry, exclusion of fluid–structure interaction (FSI) in marrow-filled cavities, and inadequate representation of age-related degeneration and healing dynamics. Moreover, technical challenges remain in linking high-resolution imaging data with subject-specific boundary conditions. To improve predictive fidelity and clinical applicability, future research must integrate poroelasticity, mechanobiological signaling, and stochastic modeling of trabecular remodeling processes.

**Table 2:** Multiscale FE studies: cancellous bone

Multiscale focus	Year	Key contributions	Limitations	References
Implant osseointegration (μCT vs. CT)	2024	Progressive implant thread modeling across scale geometries	Simplified healing-phase transitions; static loading	[81]
Viscoelastic trabecular bridging	2023	Microindentation-calibrated viscoelastic model linking micro relaxation to macro responses	Excluded plasticity; curve-fitting dependency	[83]
Homogenized 3D-printed trabecular implants	2019	Adaptive element homogenization predicts patient-specific stiffness	Neglects fatigue effects; isotropic assumption	[84]
Cortical–trabecular load sharing in distal radius	2018	Strain refinement under physiological boundary conditions	Partial volume artifacts in micro-FE	[77]
Trabecular damage characterization	2017	Non-isotropic damage behavior under multiaxial loads	Tissue assumption and interspecies variability	[82]
Multiscale model of vertebral trabecular bone	2016	Anisotropic elastic properties prediction from nano to micro levels	Limited to elastic behavior and simplified geometry	[85]
Trabecular yield surface characterization	2016	Shear anisotropy revealed through nonlinear homogenization and 160 load cases	Homogeneous assumptions; no experimental validation	[86]
Hierarchical trabecular bone properties	2015	Orthotropic property prediction from sub-nano to microscale	Excludes porosity effects; limited to orthotropic behavior	[87]

As summarized in Table 2, FE models of cancellous bone are progressively capturing the interplay between its porous microarchitecture and macroscopic mechanical response, particularly in remodeling, viscoelasticity, and implant osseointegration. Nonetheless, persistent limitations such as idealized trabecular geometry, omission of marrow-fluid coupling, neglect of fatigue and plasticity effects and restricted experimental validation indicate that further methodological refinement is essential to reflect the true physiological behavior of cancellous tissue.

### Non-FE Classical Techniques

Several classical techniques have emerged as promising alternatives to the FEM to address multiscale nature of bone behavior. Quantum mechanics (QM) functions at the atomic level and reveals insight into the behavior of the molecular constituents of bone. Although relatively new in bone modeling, QM can assess atomic interactions, such as bond formation and breaking, which are paramount to understanding growth and failure mechanisms in detail. Due to its high computational cost, QM is typically limited to smaller systems and shorter timescales, making it a useful but niche tool in modeling. In addition, classical MD and coarse-grained MD (CGMD) are particularly well-suited for practical applications at the nanoscale [88]. While MD offers detailed insight into the interactions between collagen and hydroxyapatite (HA), CGMD serves as a bridge between molecular-level interactions and continuum modeling, enabling the study of larger structures over longer timescales. Both methods represent substantial advances in our understanding of bone remodeling. Nonetheless, the computational burden remains their primary limitation [88, 89].

Meshless methods, such as the natural-neighbor radial-point-interpolation method (NNRPIM), establish another alternative to FEM, which is appropriate for modeling complex geometries. These techniques can ignore predefined meshes and manage the complex bone microstructure with much flexibility and efficiency while their development and validation still proceed [90]. Meanwhile, continuum micromechanics provides a framework for determining bone properties based on the sound microstructural rationale of a composite material with distinct phases. While the methodology is well-established, the challenge lies in accurately definition of the representative phases and capturing the complex microstructural features [91–93].

In addition, agent-based modeling (ABM) is an emerging technique that examines bone behavior at the cellular level. In ABM, individual cells interact continuously, providing real-time insights into how cellular processes of ossification and resorption translate into bone behavior at the macroscale. However, one of the major disadvantages of ABM is the high computational cost to simulate large number of agents [42, 94]. Lastly, variational multiscale methodologies (VMMs) are other advanced techniques that couple macroscopic and microscopic scales and phenomenologically assimilate many physical phenomena, such as the action of fluids or elasticity in bone tissue [95]. Although many of these models are mathematically complex, they are becoming increasingly relevant for capturing the multifaceted nature of bone behavior, thereby opening promising avenues for future research. Their integration with experimental data and incorporation into hybrid models can significantly enhance our understanding of bone biomechanics and tissue regeneration.

The recent advancements in bone modeling, as summarized in Table 3, demonstrate a shift toward increasingly sophisticated and expansive multiscale frameworks that address some of the inherent limitations of traditional finite element methods (FEM). Progress in non-FEM-based multiscale modeling has deepened the mechanistic understanding of bone behavior. For instance, hierarchical micromechanics models for cortical bone [92] and Asymptotic Expansion Homogenization (AEHG) approaches [96] have been developed to capture elastoplastic and viscoplastic responses across six orders of magnitude. These models are grounded in solid physical principles and well-defined material formulations; however, they are computationally intensive and often rely on idealized stress-state assumptions. When image-informed or orientation-based models are employed—for example, to analyze collagen alignment near implants [97] or to perform stiffness segmentation of osteons [98]—they provide higher structural fidelity and spatial resolution. Nonetheless, they are constrained by incomplete representations of mineralization patterns and dimensional simplifications. Viscoelastic and statistical models [99, 100] have incorporated time-dependent and probabilistic aspects of collagen behavior, improving the prediction of mechanical responses under dynamic loading. Still, integrating biological variability due to hydration, aging, and biochemical coupling remains a significant challenge. Similarly, stochastic models of mineralization [93] have offered new insights into tissue property variability but often simplify microstructural geometry and exclude molecular-level details.

**Table 3:** Multiscale non-FE studies associated with bone mechanics

Multiscale focus	Year	Bone type	Key contributions	Limitations	Articles
AEHG for elasto-viscoplastic bone	2024	Cortical	AEHG to model inelastic processes and strain-gradient plasticity	Requires technical assumptions; no experimental validation	[96]
Hierarchical micromechanics for elastoplasticity	2024		A fully convergent hierarchical micromechanics model for bone elastoplasticity across six orders of magnitude; a novel multiscale iterative return-mapping algorithm for defining stress states within a multiscale framework	Computationally intensive; algorithmic complexity in considering stress states	[92]
Collagen orientation in peri-implant bone	2024		Homogenization model for effective elastic properties near implants	Limited by 2D data; lacks mineralization and pore shape data	[97]
Viscoelastic modeling across three levels	2022		Three-level hierarchical model for dynamic viscoelasticity of cortical bone	Homogenization assumptions may miss hydration and aging effects	[99]
Osteon stiffness modeling	2021		Novel image segmentation method to model osteon stiffness	May not capture all microstructural variations	[98]
Statistical model of collagen fibers	2021		Statistical elasto-plastic model for collagen fibers at multiple scales	Assumes constant hydration levels	[100]
Stochastic modeling of bone matrix	2017		Stochastic model quantifying uncertainty in bone mineralization	Simplified microstructure; lacks data on collagen mineralization	[93]
Multiscale fluid-driven mechanotransduction	2018	Cancellous	Multiscale model linking pore-scale pressure gradients to bone remodeling	Assumptions on pore geometry and simplifications	[91]
Racehorse metacarpal bone adaptation	2018		Model of bone adaptation to training loads in racehorses	Refinement required in mechanosensitivity and loading conditions	[101]
Woven bone fracture healing	2017		Micromechanical model for elastic properties of woven bone	Simplified fibril organization and mineral geometry	[102]

In the context of cancellous bone, fluid–structure interaction (FSI), adaptation, and repair processes have also been investigated across scales. A pore-scale mechanotransduction model [91] linked local pressure gradients to bone remodeling via osteocyte stimulation, offering foundational insights into mechanosensing. However, the model's geometry was simplified and lacked representation of cell–matrix signaling. A related model applied to racehorse metacarpal bone [101] captured adaptive responses to athletic loading but highlighted the persistent challenges in defining mechanosensitivity parameters and accurately specifying boundary conditions. Additionally, a micromechanical model of woven bone [102] aimed at early-stage healing behavior employed geometric simplifications of both mineral and fibril structures.

In conclusion, non-FE multiscale modeling approaches exhibit a remarkable breadth and depth in both quantitative and qualitative aspects. Yet, they continue to face key limitations, including limited experimental validation, reliance on idealized assumptions, and insufficient biological realism, barriers that must be addressed to enhance their clinical and translational relevance.

In summary, Table 3 shows that non-FE multiscale approaches, including micromechanics, homogenization, stochastic modeling, and FSI-based methods, provide valuable mechanistic insights across scales and across elastic, plastic, and poroelastic regimes. While these techniques offer notable analytical and computational advantages, their broader applicability is still constrained by idealized assumptions, high computational cost, limited biological realism, and incomplete experimental validation.

### Verification and Validation Techniques

Credible FEM requires methodical verification and validation across scales. Verification of FE models in bone research is typically performed by comparing model predictions with experimental data obtained from mechanical testing of corresponding bone specimens. This involves generating an FE model from CT images of the specimen, replicating the same loading conditions experimentally, and assessing agreement between simulated and measured responses such as force–displacement curves, reaction forces, and strain or displacement fields. Statistical metrics, including correlation coefficients and root-mean-square error (RMSE), are commonly employed to quantify this agreement. A high level of concordance validates the model’s predictive capability, enabling its application for virtual testing under various conditions and thereby reducing the need for extensive physical experimentation [103–106]. Verification of FE models is also essential to establishing the credibility of multiscale simulations of bone, especially considering the hierarchical organization and complex anisotropic mechanical behavior of osseous tissue. Although an overly complex model can be difficult to validate, numerous studies have reported consistency between FE predictions and experimental measurements of strain fields, load-bearing capacities, and failure patterns, particularly in the vertebral body, femur, and trabecular bone microstructures [76, 79, 83, 107, 108]. Despite these promising outcomes, critical discussion remains necessary because most validation efforts rely on localized strain data or simplified loading conditions. Such approaches may inadequately capture the complex mechanical interactions occurring across scales or fail to reflect physiological boundary conditions [74]. Of particular importance is the verification of scale-bridging mechanisms such as those employed in homogenization schemes or two-way coupling strategies, which must ensure that microscale heterogeneity and architecture meaningfully inform macroscale predictions [57]. While some models demonstrate this level of fidelity, the field still lacks a standardized set of scale-specific benchmarks and comprehensive datasets that incorporate time-dependent, nonlinear, and remodeling behaviors of bone. The robustness of FE-based multiscale frameworks therefore hinges on four key criteria: predictive accuracy, physical plausibility, reproducibility, and sensitivity to both biological variability and modeling assumptions. These criteria must be systematically evaluated to ensure the reliability and translational relevance of any rigorous bone-modeling framework.

Among other classical methods, the NNRPIM has been validated through comparisons with traditional homogenization techniques and further examined using patch-based analyses to evaluate its accuracy and robustness at the microscale [90]. At the nanoscale, the verification of MD and CGMD simulations typically relies on internal consistency and fundamental physical principles, such as QM [88]. Although direct experimental validation at these scales is limited, MD-derived properties such as interfacial traction–separation laws are often integrated into higher-scale FE models. The subsequent agreement of these multiscale simulations with macroscopic experimental data serves as an indirect validation of the atomistic models [89]. QM simulations, particularly those based on *ab initio* approaches, are inherently verified through first-principles theory. However, practical validation arises when their outputs are embedded into larger-scale models that are benchmarked against experimental results [88]. Homogenization techniques, which are pivotal in linking micro- to macroscale mechanics, are generally validated by comparing predicted effective properties with experimental measurements, FE simulations of representative volume elements (RVEs), or analytical models such as the Mori–Tanaka and Generalized Self-Consistent Method, GSCM [92, 99, 109]. The integration of high-resolution microstructural data, such as from micro-CT imaging, further enhances the biological realism and empirical grounding of these models [93, 97]. VMMs, which couple physics across scales using variational principles, have been theoretically validated by demonstrating consistency and energy conservation across scales [24, 95]. However, experimental validation of VMMs applied specifically to bone remains limited and warrants further empirical investigation.

Overall, non‑FE multiscale approaches in bone mechanics have been extensively verified and validated through rich and systematic experimental programs, particularly within hierarchical micromechanics and homogenization‑based frameworks. Robust validation has been demonstrated across elastic, poroelastic, viscoelastic, and inelastic and strength‑related regimes [12, 92, 110–115]. Nevertheless, owing to the methodological diversity of non‑FE approaches, validation strategies remain heterogeneous and typically rely on the combination of complementary experimental techniques across molecular, tissue, and organ scales. Such multiscale validation frameworks enable reliable prediction of mechanical behavior using a wide range of experimental modalities, including ultrasonic and Brillouin scattering, electron microscopy-based hierarchical analyses, and quasi‑static mechanical and physicochemical testing [116]. This stands in contrast to the more standardized verification and validation pipelines commonly employed in image‑based finite element modeling. A comprehensive synthesis of hierarchical biomechanics models and their validation strategies is provided in [116] or interested readers. In summary, non-FE bone models are validated by demonstrating correct scale transitions and accurate prediction of effective properties across multiple hierarchical levels using independent experiments.

## Data-Driven Multiscale Techniques

The key distinction between data-driven and classical modeling approaches lies in their foundational principles. Classical methods are typically grounded in well-defined constitutive equations and physical laws, while data-driven approaches derive insights directly from experimental measurements, simulations, or imaging datasets—bypassing the need for explicit material models. This enables data-driven methods to reduce computational demands in multiscale simulations and to more flexibly accommodate noisy, sparse, or incomplete datasets [117]. The following section explores data-driven techniques that have shown promise in capturing the multiscale mechanical behavior of bone.

### Feedforward Artificial Neural Network (ANNs)

Among the most widely used ML models for bridging microscale features and macroscale responses, feedforward artificial neural networks (ANNs) stand out for their effectiveness. These models typically take inputs such as density distributions, structural parameters, and loading conditions, propagating them through one or more hidden layers to learn complex, nonlinear, and multivariate relationships, patterns that classical methods often struggle to capture [27, 117]. ANNs have been successfully applied to predict bone stiffness, Young’s modulus, failure behavior, fatigue damage, and knee joint contact forces [118, 119]. These predictions are achieved through supervised learning, using extensive input–output datasets to train the network. ANNs have been integrated with finite element analysis (FEA), where the trained network serves as a rapid surrogate model for estimating stress–strain responses demonstrated in aortic simulations [120] offering significant computational efficiency over traditional methods. This approach holds promising potential for applications in bone biomechanics as well. Training is commonly performed using backpropagation algorithms such as Levenberg–Marquardt or Bayesian regularization, which have demonstrated strong performance especially when working with sparse or noisy data [121, 122].

### Convolutional NNs (CNNs)

CNNs build upon feedforward ANNs by incorporating convolutional and pooling layers, enabling automatic extraction of hierarchical features from image data [123, 124]. In bone biomechanics, CNNs have been applied to predict the elastic modulus of trabecular bone directly from two-dimensional Dual-energy X-ray Absorptiometry (DXA) images, effectively capturing spatial patterns correlated with mechanical properties [16]. Through convolutional filtering and pooling operations, CNNs reduce noise and enhance critical structural features, facilitating tasks such as bone quality assessment, fracture detection, and high-stress region identification [124]. Furthermore, CNNs have been integrated with other computational methods for 3D material homogenization and topology optimization, allowing for scalable predictions in complex, multiphase bone tissues [125, 126]. Their capacity to learn from diverse image datasets significantly enhances the precision and scope of data-driven, multiscale investigations of bone behavior [16, 127].

### Support Vector Machines (SVMs)

SVMs have been applied to classify and predict load-bearing capacity and health status of bone by optimizing a hyperplane that separates data into distinct categories [117]. SVMs have been also used to perform bone age assessment [128, 129] and can be integrated with FEA-like approaches, where simulated outputs such as stress fields or density distributions, serve as input features for model training. Such applications have facilitated the classification of healthy versus osteopenic bone, estimation of femoral loading, and assessment of fracture risk, particularly when combined with medical imaging data [130]. Compared to ANN, SVMs are particularly well-suited for problems involving small to moderate datasets, where interpretability, robustness, and generalization are crucial [131]. Despite these strengths, ANNs have demonstrated higher accuracy than SVMs in predicting proximal femur loads from bone geometry and density distributions [132], suggesting that model choice should depend on data availability and the specific predictive task. Although not yet used, the effectiveness of SVMs in solving classification tasks can potentially make this method a valuable tool for multiscale bone research [117].

### Self-Organizing Map (SOM)

Although less commonly applied in bone mechanics compared to supervised learning methods, SOMs offer a powerful unsupervised learning approach for pattern recognition in high-dimensional data. SOMs function by projecting complex, unlabeled data onto a low-dimensional topological map, revealing hidden structures and relationships. This is particularly valuable for analyzing multidimensional biomechanical data from gait analysis to identify distinct movement patterns [133]. Heterogeneous bone-related datasets that span multiple scales can be potentially analyzed using this method. Early applications of SOMs in bone research include image-based analysis of bone structures [134] and time-based classification of fracture healing stages and clustering of microarchitectural parameters to uncover emerging patterns in bone quality and structure [135]. By identifying unusual clusters or suggesting alternative groupings, SOMs can facilitate hypothesis generation and potentially reveal correlations with mechanical properties [136]. Therefore, SOMs could offer a probing pathway into the multiscale complexity of bone, complementing traditional analytical and supervised modeling techniques.

### Clustering

The analysis of multiscale datasets of bone involves methodology such as porous microstructure structural information mapping (SIM), which involves using classical algorithms, including *k*-means and hierarchical clustering. Clustering uncover intrinsic patterns within mechanical and morphological variables by grouping observations without the need for labeled datasets [137, 138]. In the context of bone mechanics, clustering has been used to classify microstructural features, segment large-scale simulation outputs for efficient post-processing, and even guide treatment planning by grouping patients who exhibit similar patterns of bone degradation [139]. This approach enables a deeper understanding of underlying structural differences and can reveal associations between microarchitectural characteristics and mechanical performance. Furthermore, when applied to FEA results or imaging data, clustering often aligns with regions of high strain or similar microarchitectural changes. Such methodologies offer a novel lens through which the convergence of multiscale factors influencing bone health can be explored potentially improving both diagnostics and therapeutic strategies [140].

### Principal Component Analysis (PCA)

PCA is a multidimensional analysis tool used in multiscale bone research. It facilitates the transformation of high-dimensional, often highly correlated variables—such as CT-derived geometries, strain distributions, and morphological metrics—into a smaller set of uncorrelated principal components that capture the majority of the dataset’s variance [141]. By isolating the most informative components, PCA reduces computational complexity and enhances interpretability, helping researchers focus on the dominant factors influencing bone mechanics. This makes PCA particularly suitable for population-based studies, where large, heterogeneous datasets involving anatomical, mechanical, and clinical parameters must be compared and analyzed collectively. Moreover, PCA provides an unsupervised analytical framework to investigate how local tissue properties and global structural geometry interact across spatial scales. For instance, it has been used to identify key anatomical variations in the femur that correspond to different loading conditions or fracture risks [130, 141].

### Data Fusion

Data fusion involves integrating inputs from multiple modalities such as imaging, mechanical testing, and simulation outputs to construct comprehensive multiscale models of bone behavior. ML techniques are well-suited for merging heterogeneous datasets (e.g., density maps, strain fields, and clinical indicators) into unified predictive frameworks [142]. By leveraging multi-fidelity data sources, researchers can address hierarchical knowledge gaps and improve predictions of both local tissue properties and global structural behavior. This integrative approach plays a critical role in advancing personalized medicine, wherein patient-specific imaging and diagnostic data can be embedded within broader population-based models or simulation workflows. Ultimately, data fusion enables real-time clinical decision-making and paves the way for individualized treatment strategies in multiscale bone biomechanics [143, 144].

### Recent Contributions from Data-Driven Techniques

Recent advances in data-driven techniques have led to a well-defined taxonomy of research directions, methodological innovations, and application domains in the multiscale modeling of bone, as summarized in Table 4. Broadly, these studies fall into three interrelated themes: (i) structure–property prediction frameworks, (ii) modeling of bone adaptation and regeneration, and (iii) hierarchical elasticity estimation across scales.

**Table 4:** Multiscale modeling of bone: Data-driven studies

Multiscale focus	Year	Bone type	Key contribution	Limitations	Articles
Whole vertebra: unified stiffness via density–modulus fit	2025	Cortical/cancellous	Data-driven density–modulus model via micro-CT + DVC + QCT + FEA	Small elderly sample; isotropic assumption; lacks anisotropy/fabric; needs clinical validation	[145]
Statistical inverse modeling (macro–meso)	2021	Cortical	ANN-based solution for identifying stochastic elastic field parameters using simulation and DIC data	Simplified 2D problem; uncertainty quantification in experiments excluded	[153]
Micro-to-macro via DIC + data-driven solver	2020		Multiscale elasticity prediction using experimental data	Needs stress estimation; limited to postprocessing	[152]
Full musculoskeletal (muscle–bone) system	2016		EMG-informed multiscale modeling of muscle–bone interaction	High computational cost; complex data integration	[141]
MCM → MCF → MCFR (nano hierarchy)	2016		FEA + NN to predict elastic properties across nanoscale levels	Simplified assumptions; ignores NCPs and hydration; limited to elastic regime	[148]
Fibril-level elasticity modeling	2015		FEA + ANN to estimate effective fibril properties	Limited to fibril scale; no integration into full multiscale workflow	[118]
Macro–micro FE + ANN for site-specific bone ingrowth	2024	Cancellous	FEA + ANN to predict implant-site bone ingrowth	Single CT case; static loading; limited ANN training dataset	[146]
RVE + ANN for scaffold mechanical optimization	2023		ANN optimization based on FEA of porous dental scaffold with varying TiO_2_ content	Specific to Polyvinylpyrrolidone (PVP)/Alginate–TiO_2_; limited to tensile loading	[147]
Inverse ANN modeling of cancellous bone	2022		ANN predicts volume fraction of cortical bone from magnetic field simulation inputs	Simplified cylindrical geometry; idealized field input; lacks real imaging data	[150]
Microstructure-based stiffness via plate/rod metrics	2022		Probabilistic descriptors of trabecular architecture	Sample-specific; only femoral cubes; linear regression	[154]
DXA-based image; trabecular elasticity	2021		CNN model predicting elastic modulus from simulated DXA images	Simulated DXA input; limited donor set; constant tissue properties	[16]
Scaffold regeneration modeling	2021		ML-based prediction of bone formation in scaffolds	Focus on late-stage healing; scaffold degradation not modeled	[149]
Strut-level network modeling of trabecular bone	2019		Graph + beam model for trabecular structure and stiffness	Linear beam model; lacks failure simulation	[151]

A prominent body of work employs ANNs and CNNs to predict elastic moduli or bone ingrowth patterns using image-based data (e.g., DXA, micro-CT) and FEA-generated data [16, 145–148]. For example, deep learning applied to simulated DXA images has outperformed traditional histomorphometric regression in predicting trabecular bone elasticity [16], while ANN models of the bone–implant interface have significantly reduced the computational burden for site-specific ingrowth prediction [146]. Another notable trend is the integration of RVEs and mechanoregulatory criteria into ML pipelines. These frameworks simulate localized remodeling, scaffold optimization, and probabilistic behavior under uncertainty [147, 149–151]. When validated against *in vivo* or *ex vivo* data, such models have demonstrated high predictive accuracy while substantially reducing computational costs, particularly in the context of bone tissue engineering and regenerative medicine [149]. A third research direction focuses on inverse modeling and statistical identification techniques to estimate mesoscopic material properties from macroscale observations. For instance, digital image correlation (DIC) has been coupled with probabilistic neural inference to solve high-dimensional inverse problems, such as reconstructing cortical bone elasticity fields [152, 153].

More recently, efforts have shifted toward whole-organ integration by combining Quantitative Computed Tomography (QCT)-FEA with digital volume correlation (DVC), enabling density and modulus mapping across both cortical and trabecular regions of the vertebral body [145]. At the nanoscale, hierarchical elasticity modeling using FEA–ANN hybrid approaches has been developed to predict mechanical responses at the collagen microfibril and fibril levels, reflecting the increasing complexity and resolution of multiscale surrogate models [118, 148]. Despite these advancements, several challenges persist. Limitations include overly simplified anisotropy assumptions [145], limited anatomical generalizability [146, 150], absence of failure or damage modeling, and lack of feedback mechanisms for remodeling [148, 149]—all of which hinder clinical translation. Nonetheless, continued progress in deep learning surrogates, multimodal data fusion, and the development of digital twins of skeletal tissue points toward a promising future for scalable, interpretable, and biologically informed multiscale models of bone mechanics and adaptation.

Table 4 highlights the growing expansion of data-driven methodologies capable of predicting multiscale bone properties, remodeling behavior and structure–property relations across scales with substantial computational efficiency. Nevertheless, many models still face challenges related to simplified anisotropy, limited anatomical generalizability, and the absence of explicit mechanobiological feedback as well as their dependence on training datasets. These constraints indicate the need for more integrated and physics‑aware ML frameworks.

### Verification Techniques

ML-based frameworks are typically trained on high-fidelity numerical simulations or experimental datasets and validated through a combination of statistical, comparative, and biological approaches. The ANNs are often validated by comparing their predictions—such as stress–strain responses, strain energy densities, and evolving bone density distributions—with outputs from multiscale FE models, as well as *in vivo* animal studies or clinical imaging data. These validations include both quantitative metrics (e.g., mean absolute error, *R*^2^ scores) and qualitative indicators, such as consistency with radiographic patterns of adaptation or degradation [155, 156]. Similarly, CNN-based homogenization models are evaluated by comparing their predicted effective mechanical properties on unseen microstructures with analytical models or high-resolution FE simulations [157]. PCA models, when used for anatomical and density variation prediction, have demonstrated high fidelity through low residual errors between reconstructed and actual femoral geometries and density maps [158, 159]. SOM have been applied in unsupervised contexts to map biomechanical determinants of bone remodeling, with validation achieved through alignment with patient-specific imaging data, such as cone-beam computed tomography [160]. Across all methods, validation strategies typically rely on both internal consistency via dataset fusion or simulation coherence, and external validation against empirical or gold-standard simulation benchmarks. Critical to the credibility of these data-driven models is the use of cross-validation, error analysis, and longitudinal correlation with *in vivo* outcomes. However, to elevate these frameworks to the mechanistic rigor of classical modeling techniques, greater emphasis must be placed on interpretability, uncertainty quantification, and the integration of biophysical constraints. Ultimately, while ML techniques offer considerable benefits in terms of computational efficiency and scalability, validation must extend beyond predictive accuracy. Ensuring biological relevance, cross-scale consistency, and robustness under physiological variability is essential for their successful translation into clinically meaningful applications.

## Hybrid Multiscale Techniques

The term “hybrid” is today most commonly associated with ML‑enhanced frameworks, however, the underlying concept long predates the advent of ML. Classical physics‑based bone biomechanics has, for decades, relied on multiscale hybrid strategies that combine different frameworks. Examples include multiscale coupling of CT/X‑ray physics, micromechanics, and FE modeling to derive subject‑specific material properties and structural responses across scales, as demonstrated in previous works [8, 161, 162]. Likewise, hybridization between micromechanics and systems‑biology‑based remodeling formulations has been established to connect tissue‑level mechanical stimuli with cell‑mediated adaptation [18, 163]. These classical hybrid approaches have played a central role in linking structural, compositional, and mechanobiological processes across scales and laid the foundation for current physics‑enhanced ML frameworks by demonstrating the value of cross‑scale and cross‑theoretical coupling long before data‑driven paradigms emerged. In this review, however, our focus is on emerging data-driven, ML-enhanced hybrids that integrate physics‑based modeling with data‑driven ML techniques. This distinction allows us to clarify the conceptual evolution while highlighting the recent methodological shifts enabled by ML.

Conventional ML models such as deep NNs and ensemble methods typically operate within a descriptive framework that often overlooks the underlying physical context. While these models can achieve high accuracy in capturing complex relationships between input parameters and outputs, they usually require large labeled datasets and provide limited assurance that their predictions comply with known physical laws. As a result, they may produce non-physical outcomes, particularly when extrapolating beyond their training domain, and often function as black boxes with limited interpretability [164–166]. To address these limitations, hybrid approaches combining classical physics-based modeling with data-driven methods have gained prominence. A study with a hybrid FE-ANN approach for site-specific prediction of bone ingrowth around a porous-coated ankle implant investigated the patient-specific CT-based modeling of the implanted tibia (macroscale) to quantify implant-to-bone micromotion, microscale FE simulation of bone ingrowth using a mechanoregulatory algorithm, and a trained ANN. This study aimed to rapidly map micromotion metrics to predicted ingrowth outcomes and presents a clinically translatable and computationally efficient workflow that combines classical FEA with data-driven surrogate modeling to assess osseointegration potential [146].

In addition, physics-based machine learning (PBML) has emerged as a compelling paradigm that integrates governing physical principles, such as partial differential equations (PDEs), boundary conditions, and conservation laws, directly into neural network architectures or loss functions [167, 168]. This integration enables models to learn from data while complying with physical and biological laws, a key requirement for accurate modeling of complex materials like bone. A prominent example is physics-informed neural networks (PINNs), which incorporate governing partial differential equations (PDEs) such as those in elasticity or poroelasticity and boundary conditions into their loss functions through automatic differentiation. This enables the realistic simulation of mechanical responses across multiple hierarchical levels from nanoscale to macroscopic deformations even when only limited or noisy data are available [169–172].

To enhance multiscale modeling fidelity, higher-order multiscale PINNs (HOMS-PINNs) have been recently introduced, offering a more effective bridge between micro- and macro-scale bone mechanics. Additionally, theory-constrained PINNs (TCPINNs) embed domain-specific theoretical constructs such as damage mechanics or bone remodeling laws directly into the network architecture, thereby improving both predictive accuracy and biomechanical interpretability [156, 167, 173]. Despite their promise, several challenges remain. Training PINNs is often computationally intensive and may suffer from optimization instability due to conflicting loss components (e.g., data fidelity vs. physical constraints) [174]. Furthermore, mature methods for modeling the nonlinear, time-dependent processes driving bone adaptation and degeneration remain lacking.

Physics-informed neural operators (PINOs), an operator-based generalization of physics-informed neural networks (PINNs), address some of the limitations mentioned earlier by learning mappings between functional spaces rather than pointwise solutions. This makes them particularly suitable for parameterized geometries and heterogeneous material distributions, as commonly encountered in bone mechanics (e.g., patient-specific CT-based models). PINOs enable fast, mesh-free predictions across varying conditions, such as gait analysis or implant planning [175, 176]. Additionally, mechanics-informed machine learning (MIML) incorporates physical constraints through feature engineering, customized loss functions, or hybrid neural-mechanical architectures. Another promising framework is thermodynamics-informed neural networks (TINNs), which enforce constraints such as energy conservation and entropy production. This makes them appropriate for modeling biological processes [177]. However, these advanced frameworks often require complex domain-specific formulations, careful hyperparameter tuning, and sufficient validation data. While ongoing research continues to refine and expand these approaches, they remain part of an emerging field with significant potential for improving scalability, interpretability [178], and physical consistency potentially suitable for models aiming to represent bone’s hierarchical nature.

**Fig. 3:**
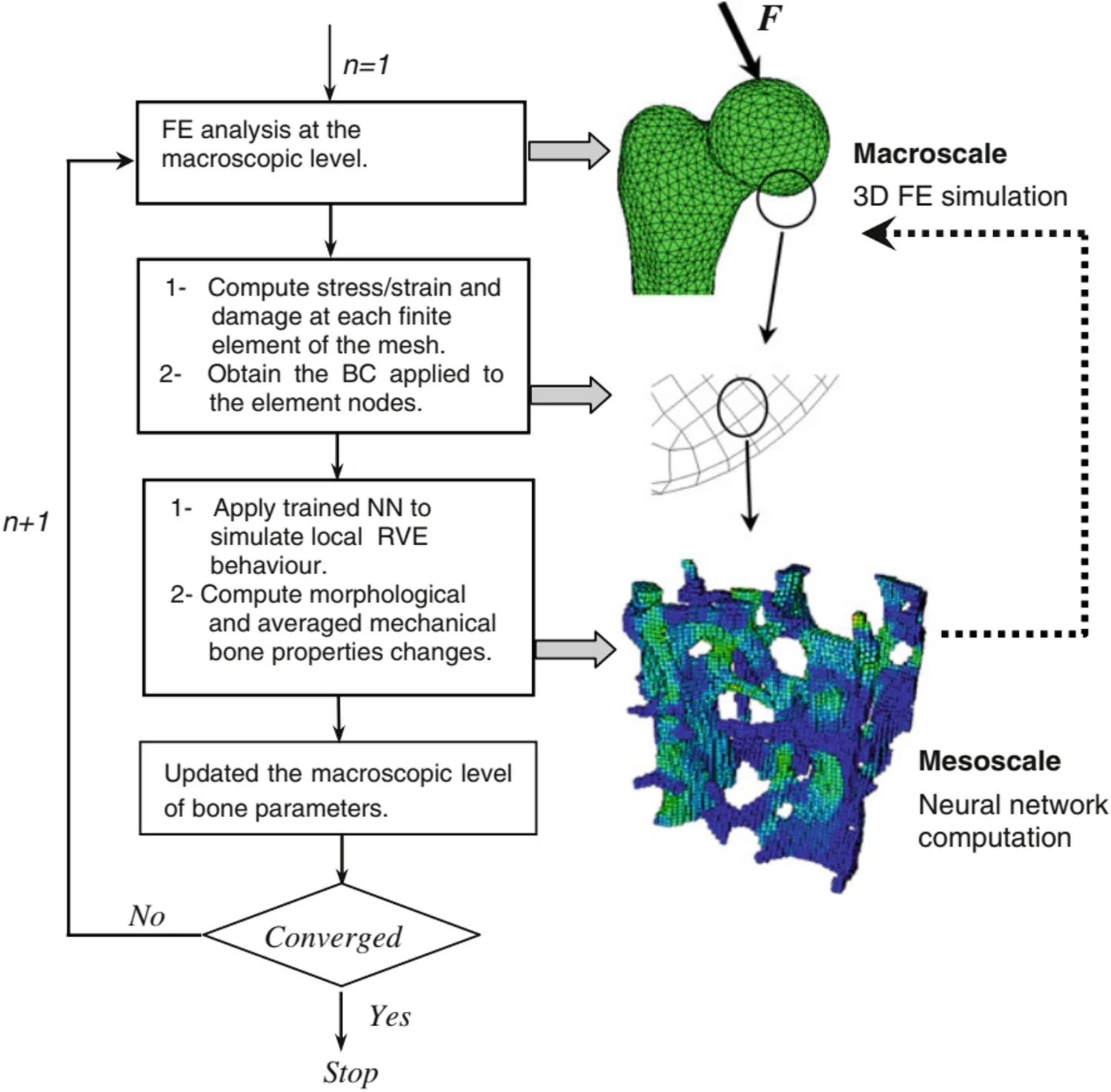
Multiscale hybrid FE-NN framework for assessing bone: A dual-level evaluation to forecast the remodeling process. FEA of bone at the macroscale and NN computation at the mesoscale (adapted with permission from [179])

Advanced hybrid modeling approaches, particularly those grounded in PBML, have improved the integration of mechanistic FE frameworks with data-driven algorithms. Unlike purely data-driven models, which often rely on empirical correlations and may lack fidelity to governing physical laws, PBML techniques maintain mechanistic consistency while mitigating the high computational cost of conventional simulations. Although hybrid models are not yet widely applied in the field of bone biomechanics, a few studies have begun to explore their potential in this context. While further research is still needed, these initial efforts highlight the promising capabilities of such approaches [146, 156, 179]. As shown in Fig. 3, a macro-micro-FE-ANN framework was developed to predict implant-site bone ingrowth. In this model, site-specific micromotion inputs derived from FE analyses were used to train an ANN, which emulated mechanoregulated bone formation outcomes. This model was validated by comparing mesoscale predictions with conventional FE^2^ outputs and qualitative trends observed in radiographic imaging [179]. This approach achieved a substantial reduction in simulation time while preserving biomechanical accuracy. Similarly, a multiscale ML-based framework for scaffold-mediated bone regeneration replaced computationally intensive nested FE^2^ (two-scale FE) calculations with a hierarchy of neural networks. These networks were trained on homogenized elastic responses and strain energy densities from RVEs, while remodeling dynamics were preserved using inverse parameter identification informed by *in vivo* data [156]. These examples demonstrate the principles of PBML, where neural networks function not as opaque black-box models but as physics-informed computational surrogates. By shifting from black-box prediction to interpretable, efficient, and physically reliable simulation, these well-constructed hybrid frameworks illustrate the powerful synergy between ML generalization and FE structural fidelity. This consolidation strengthens predictive robustness across scales and establishes a foundation for transferable, scalable frameworks that can accelerate *in silico* modeling in bone biomechanics.

### Verification Techniques

The classical validation of hybrid modeling approaches typically involves comparison against analytical solutions, direct numerical simulations (e.g., FEM) [179], and experimental or even *in vivo* data, depending on the application domain. These validation strategies help ensure that the integration of data-driven components does not compromise physical accuracy or predictive reliability. For PINNs and PINOs, verification techniques are grounded in methodologies such as residual minimization of governing PDEs, convergence studies, and generalization to previously unseen conditions [180].

TCPINNs and TINNs incorporate specific thermodynamic principles such as energy conservation and entropy production, which enable the modeling of complex material behaviors, including viscoelastic dissipation [177]. These built-in theoretical checks ensure that the learned representations remain consistent with fundamental physical laws, enhancing reliability in modeling time-dependent and irreversible processes. In MIML, techniques such as stress decomposition and data-driven surrogate modeling are employed to preserve mechanical interpretability and uphold established formulation laws. These strategies also facilitate efficient basis scaling, particularly in multiscale biomechanical simulations [179]. Despite these advancements, challenges such as ensuring the consistency of boundary conditions, implementing robust uncertainty quantification, and aligning model predictions with the biological variability inherent in tissues such as bone remain. Therefore, the adoption of PBML in real-life biomechanical applications will require rigorous, multi-faceted validation frameworks encompassing physical fidelity, statistical accuracy, and biological relevance [173, 179].

## Potential Applications of Classical, Data-Driven and Hybrid Models

Classical multiscale modeling techniques, particularly FEM, remain the powerful tool for translating multiscale insights in bone mechanics into clinically and experimentally actionable predictions and continue to play a central role in advancing bone health assessment, fracture risk prediction, optimization, and personalized therapeutic planning [181, 182]. FEM remains uniquely valuable because it can incorporate patient-specific geometry, heterogeneous material properties, and multiscale mechanical phenomena across scales. Notably, QCT and HR-pQCT-based FE models provide spatially resolved estimates of stiffness, failure load, and risk critical regions, thereby enabling individualized diagnostics and treatment planning compared to traditional densitometric assessments such as DXA. Multiple studies show that QCT FEA outperforms DXA for vertebral strength prediction, while nonlinear micro-FE analyses derived from HR-pQCT enhance fracture risk assessment at peripheral sites [181–185]. These patient-specific FE frameworks allow the simulation of realistic physiological boundary conditions and provide mechanistically grounded assessments of fracture risk that extend beyond density-based metrics. Micro-CT-based micro-FE derived from high-resolution imaging further serves as a “virtual test bench” to interrogate how microarchitecture and material assumptions control load transfer, whole bone failure pathways and local strain concentrations [185]. Such models offer improved diagnostic insight compared with macroscopic estimations and support population-specific or disease-specific characterization of mechanical weakness [185]. In addition to diagnostics, Micro-CT-based multiscale FEM frameworks are increasingly applied to examine bone–implant interactions by capturing implant surface topology, thread design, and local micromotion that help to predict osseointegration performance and guide implant design decisions [81]. Furthermore, coupled multiphysics FE models such as those integrating electromechanical, flexoelectric, and magnetoelectric responses provide novel opportunities for early detection of osteoporosis and for exploring diagnostic markers rooted in microstructural electromechanical behavior [15, 70–72, 74, 75]. Collectively, these advances position FEM as the most mature, physics grounded route to clinical translation among classical approaches.

Despite these advances, widespread clinical adoption remains constrained. First, computational cost remains substantial for high-fidelity micro-FE or FE^2^ workflows, necessitating reduced order or predictor–corrector strategies to reach clinically acceptable turnaround times [107]. Second, material and structural uncertainty (heterogeneous anisotropy, viscoelasticity, damage, and hydration) is imperfectly captured from routine imaging. This motivates the requirement for high-resolution imaging to extract clinically relevant microstructural features and standardized validation protocols across imaging platforms for parameter inference and uncertainty quantification [93, 95, 107]. Third, physiology realism, including marrow FSI/poroelastic effects and mechanobiological feedback requires tighter coupling to remain true to *in vivo* boundary conditions [91, 93]. Finally, generalizable validation datasets spanning ages, anatomical sites, and disease states are still scarce, which constrains credible extrapolation [107]. Addressing these gaps will directly increase the translational readiness of FEM. Beyond FEM, homogenization techniques offer practical solutions for linking microstructural data with macroscopic bone responses, particularly when micro-FE simulations are computationally prohibitive. These strategies enable estimation of effective tissue level elastic properties and allow microstructural characteristics to be embedded into coarser, organ level models [24, 93, 95]. However, broader application still depends on improving the reliability of microstructural information extracted from routine clinical imaging [93, 95]. Techniques such as MD, QM, ABM, NNRPIM, and meshless methods contribute valuable mechanistic insight at fine spatial scales [42, 88–91, 94, 97, 98], but remain computationally intensive and are therefore used primarily for basic research rather than clinical integration. Ultimately, the future of multiscale modeling in clinical practice depends on overcoming current computational and validation barriers. Continued targeted research and methodological innovation will be essential to translate these technologies into practical, scalable clinical solutions.

Emerging data-driven technologies, particularly ANNs, have been showing substantial promise in accelerating multiscale bone analyses. When trained on high-fidelity FE datasets, ANNs can rapidly estimate fracture loads, strain distributions, or remodeling stimuli, significantly reducing computational runtime while maintaining accuracy [117, 146–148]. These surrogate models facilitate real time or near real time predictions, which may prove valuable in intraoperative workflows or rapid diagnostic assessment. Deep learning techniques such as CNNs further enhance predictive capability by extracting structural information directly from DXA or CT images to estimate trabecular stiffness, detect microarchitectural deterioration, or classify tissue regions [16, 123–127]. These capabilities contribute to improved fracture risk assessment and precise tissue segmentation [16, 117, 139]. Complementary approaches such as SOM, SVMs, and PCA, and multimodal data fusion provide additional tools for pattern recognition, dimensionality reduction, and integration of heterogeneous clinical datasets, e.g., [128, 129, 131–135, 137, 140], ultimately enhancing fracture healing classification, projecting musculoskeletal loading, and generating personalized bone morphologies. In addition, data fusion techniques, which integrate diverse datasets within ML frameworks, enhance model robustness and clinical relevance, representing a critical future direction for personalized bone healing and regeneration strategies [117, 123].

Hybrid modeling frameworks that combine physics-based representations with data-driven efficiency represent a promising direction for reconciling interpretability with computational performance. For example, hybrid FE-ANN workflows have been used to accelerate patient-specific predictions of bone ingrowth around implants while maintaining biomechanical fidelity [146, 179]. These approaches integrate FE-derived micromotion metrics with ANN-based surrogate modeling to capture mechanoregulated bone formation more efficiently. These models show strong potential for the rapid simulation of patient-specific bone remodeling, leveraging local bone morphology and mechanical responses to support timely and personalized clinical risk assessment, prediction of fracture risk and treatment planning [179]. In conjunction with hybrid FE-NN modeling, PINNs and related paradigms introduce governing mechanical laws directly into the learning process, enabling data efficient modeling even when experimental or imaging data are sparse or noisy [166, 169–173]. Although PINNs are still emerging in bone biomechanics, their ability to incorporate elasticity, poroelasticity, or remodeling equations into the loss function suggests substantial potential for future multiscale simulations [166, 170]. In summary, FEM increasingly underpins hybrid workflows. FE generated fields train ANN surrogates for rapid “near real time” inference (e.g., ingrowth prediction, remodeling stimuli), while physics-informed models constrain learning to mechanically admissible solutions [146, 156, 166]. PINNs and PINOs offer mesh free or operator level acceleration for parameterized geometries and heterogeneous tissues, promising scalable uncertainty aware surrogates for clinical deployment [170, 175, 176]. In parallel, data fusion frameworks that integrate DVC/QCT observables with FE fields are enabling unified density–modulus maps for whole vertebrae, albeit with clear needs for anisotropy and larger clinical validation [145]. The message is that FEM supplies the mechanistic substrate—objective functions, boundary condition realism, and interpretable fields—on top of which data-driven components can safely accelerate computation without sacrificing physical plausibility.

Looking ahead, continued integration of classical FEM, data-driven approaches, and physics-informed ML is likely to enable more accurate, computationally efficient, and personalized assessments of bone health. FEM will remain foundational due to its mechanistic fidelity and ability to incorporate multiscale structural information, while ML will contribute scalability and rapid evaluation. The convergence of these approaches supported by advances in imaging, validation strategies, and hybrid modeling promises to enhance both scientific understanding and clinical management of bone mechanics.

## Discussion

Multiscale modeling of bone requires a careful balance between physical fidelity, data availability, computational cost, and biological interpretability. Depending on the scale of interest, model complexity, and intended application, two dominant modeling paradigms, classical physics-based and data-driven approaches, offer distinct strengths and limitations. Among classical methods, FEM remains the cornerstone of bone mechanics modeling due to their strong theoretical foundation, accuracy, and versatility across scales—from nanoscale mineral–collagen interactions to whole-bone loading scenarios [54, 56]. Its integration with medical imaging further enables patient-specific modeling for diagnosis, treatment planning, and surgical simulation, reinforcing its clinical relevance.

At the tissue and organ levels, multiscale FEM has been instrumental in elucidating load sharing and structural anisotropy. Studies resolving cortical–trabecular interactions demonstrate that boundary condition realism critically governs predicted strain distributions; oversimplified constraints can bias conclusions regarding which compartment is most failure-prone [77, 108]. These findings underscore the necessity of aligning experimental loading protocols with micro-FE boundary prescriptions when inferring patient-specific vulnerability. Age-related changes in regional mechanics have also been clarified through coupled macro–micro-FEM. Two-way simulations reveal that aging substantially amplifies cortical strains and strain energy densities at clinically vulnerable sites such as the femoral neck (often by several fold) providing a mechanistic explanation for disproportionate fragility despite modest changes in apparent density [78]. Notably, these trends persist even under idealized assumptions regarding lacuna-canalicular geometry and muscle forces, supporting targeted intervention strategies in regions where multiscale stress amplification is expected. At finer scales, FEM has advanced understanding of damage initiation and crack propagation. Bottom-up osteonal models that couple lamellar interactions capture brittle damage onset and predict crack deflection across cement lines, while fibril- and lamella-level formulations reveal how collagen–mineral topology and orientation govern crack path selection and arrest mechanisms [58, 79]. Together, these studies explain why bones with similar mineral density can exhibit markedly different failure modes, highlighting the importance of hierarchical architecture in risk stratification.

FEM has also played a central role in implant design and osseointegration planning. Multiscale simulations of threaded dental implants reveal how thread morphology influences local strain stimuli and predicted bone ingrowth trajectories, identifying design parameters that modulate peri-implant mechanoregulation [81]. Homogenization-assisted FEM applied to additively manufactured trabecular metals enables patient-specific stiffness matching at manageable computational cost, although fatigue behavior and anisotropy remain active areas for development [84]. More recently, coupled macro–micro-FEM augmented with artificial neural network surrogates has enabled rapid, site-specific prediction of bone ingrowth around porous coatings, achieving clinically relevant runtimes without sacrificing mesoscale fidelity [146]. Collectively, these efforts illustrate a continuum from detailed micro-FE interface mechanics to accelerated surrogates capable of informing preoperative and intraoperative decision-making.

Beyond mechanics alone, FEM is especially suited for capturing coupled physical phenomena such as electromechanical and poroelastic responses, which are central to the multifaceted nature of bone behavior. Electromechanical formulations incorporated into multiscale FEM (e.g., isogeometric and micromorphic implementations) suggest that strain-gradient-driven electrochemical cues may regulate osteocyte apoptosis and repair, while crack-tip electric field predictions point to potential healing-relevant biomarkers [70, 73, 74]. Magnetomechanical and magnetoelectric FEM frameworks further link porosity-dependent signals to non-invasive diagnostic strategies for osteoporosis, offering a physics-based bridge between microstructural alterations and clinically measurable markers [15]. These efforts extend prior reviews on bone piezoelectricity by proposing concrete FEM workflows for therapeutic stimulation paradigms [71, 72].

Nevertheless, FEM has its drawbacks such as high computational cost at fine scales limiting its clinical scalability, and persisting difficulties in reliably extracting bone’s heterogeneous, nonlinear, and time-dependent properties from clinical imaging data [68, 78]. Furthermore, micro-FE approaches are known to exhibit significant numerical sensitivity [161]. Mesh-free alternatives like NNRPIM offer faster modeling of complex geometries but may struggle to capture material nonlinearities with the same fidelity [186]. At the molecular level, methods such as MD, CGMD, and QM provide atomistic insights into material behavior [187], though their computational cost renders them impractical for tissue-level applications. Homogenization approaches (e.g., asymptotic homogenization, variational multiscale methods, and analytical multiscale micromechanics) and ABM have in fact been demonstrated to accurately capture bone’s anisotropy and heterogeneity studies and offered more efficient meso-to-macro scale modeling [13, 25, 95, 96, 99, 188]. Analytical homogenization approaches linking collagen scale properties to trabecular behavior have been shown to achieve accuracy comparable to micro-FE simulations while offering several orders of magnitude greater computational efficiency [188]. While certain implementations may rely on idealizations or simplified microstructural descriptions, these methods remain among the most robust tools for linking bone’s hierarchical structure to its mechanical adaptation across scales. Classical methods offer mechanistic transparency and alignment with physical laws; however, they face challenges in adaptively modeling dynamic biological processes such as bone remodeling and adaptation.

Data-driven modeling adds a complementary paradigm by using ML techniques to identify patterns from experimental or simulated datasets that can serve as rapid surrogate models with lower computational costs once trained [143, 189]. Common algorithms such as ANNs are applied for property prediction, tissue classification, and scaffold optimization [146, 147, 150, 153] and are particularly effective at capturing complex nonlinear relationships that classical methods struggle with [120]. However, their lack of interpretability and physical grounding limits their generalizability beyond training data [190, 191]. CNNs, on the other hand, are well-suited for extracting mechanical properties from medical images such as DXA or CT, making them useful in diagnostics and personalized modeling [16, 123, 124, 148]. Their ability to integrate diverse clinical datasets enables real-time patient-specific predictions and population-level studies. Unsupervised methods like PCA and SOMs are valuable for dimensionality reduction and data visualization, though less useful for predictive tasks [130, 135, 141]. Despite their strengths, ML models often lack physical constraints, which poses a challenge for clinical reliability and mechanistic validity. Data fusion architectures can capture hierarchical interactions from microstructure to macroscopic behavior [192].

ML success in image recognition is undeniable, with immediate diagnostic applications made possible by access to large volumes of annotated data. However, it often performs less effectively in prognostic tasks, particularly when data are sparse. In such cases, traditional physics-based simulations continue to play an indispensable role. Therefore, hybrid strategies like PBML address this gap by embedding physical laws, governing equations, or multiscale principles into ML frameworks, enhancing both interpretability and biomechanical consistency. A notable example is PINNs, which incorporate governing PDEs (e.g., elasticity, poroelasticity) directly into their loss functions. This enables data-efficient, mesh-free modeling of complex structures like trabecular and cortical bone [169–172, 193]. Higher-order and multiscale variants (e.g., HOMS-PINNs) have shown promise in linking microscale material behavior to macroscale responses, supporting more comprehensive simulations. Neural operators like PINO extend this paradigm by learning solution operators over function spaces, allowing rapid generalization across geometries and material properties [175, 176]. Recent developments such as TCPINNs and MIML improve model reliability by enforcing constitutive laws (e.g., damage mechanics or bone remodeling) [156, 167, 194]. TINNs further embed principles like energy conservation and dissipation, enabling realistic simulation of complex processes such as fracture healing or mineralization [177, 195, 196]. While PBML approaches face challenges in balancing multiple loss terms, training stability, scalability, and heterogeneous patient-specific biological complexity, their ability to unify physical rigor, computational efficiency, reducing the need for extensive training datasets and interpretability positions them as a promising framework for the future of multiscale bone modeling.

As discussed, the two primary modeling paradigms have shaped the foundation of multiscale analysis in bone biomechanics: classical modeling, predominantly based on FEM [66, 67, 76, 81, 83] and related numerical techniques [88–92, 96], as well as data-driven modeling, which leverages ML algorithms [16, 145–147, 149, 150, 154]. Each approach offers distinct advantages and limitations, particularly regarding clinical applicability, computational efficiency, and scientific rigor. Both paradigms are capable of simulating bone’s mechanical responses with high fidelity across scales. Their hierarchical modeling capabilities provide insights into multiphysics mechanobiological processes that are directly relevant to clinical prediction and intervention [67, 192, 197]. Given the complementary strengths and limitations of FEM and ML, the emergence of hybrid approaches represents a logical and promising next step, offering higher interpretability, physical consistency, and improved generalizability particularly in contexts involving sparse or noisy clinical data [175, 176, 179, 193]. Despite their potential, the implementation of hybrid models remains challenging. Key issues include balancing computational efficiency and physical accuracy, defining appropriate physics-informed constraints, and ensuring scalability to complex, heterogeneous biological structures.

To guide the selection or combination of modeling strategies, this review introduces a structured decision matrix (Table 5), comprising eight criteria: multiscale scope, type of behavior, data availability, modeling goals, interpretability, computational constraints, biological realism, and speed. This matrix serves as a flexible and evolving framework to support evidence-based decision-making in research and clinical modeling. It lays the groundwork for integrating diverse modeling techniques into unified, scalable, and clinically relevant frameworks for bone biomechanics. The matrix categorizes techniques into classical, data-driven, or PBML approaches and highlights key opportunities for cross-method integration. For example:

- Nonlinear fracture behavior, where data is limited, can be effectively addressed using PINNs or TCPINNs.
- Combining FEM with MIML enhances interpretability without sacrificing modeling flexibility.
- CNNs integrated with image-based FEM are particularly effective for patient-specific applications.
- ANNs and Data-driven Multiscale Networks (DMNs) trained on high-fidelity FE datasets offer efficient surrogate models suitable for real-time simulations.

**Table 5:** Decision matrix for selecting appropriate multiscale techniques for capturing bone behavior based on various criteria

Criterion	Key questions	Suitable techniques to use	Quantitative range/benchmark	References
Scale of interest	What spatial scale is critical (nano/micro/macro/multiscale)?	µFE, Homogenization, FE^2^	Resolution 1 µm to 5 mm	[24, 156, 179]
Physical behavior	Is bone behavior linear, nonlinear, or coupled (fracture, remodeling)?	If nonlinear, choose MIML, TINNs, TCPINNs, nonlinear FEM	Accuracy should be more than 85%, RMSE should be less than 0.08	[152, 156, 179]
Data availability	Is high-quality, multiscale data available?	CNN, PCA, data fusion	Dataset size 150–2500 samples	[16, 145, 158]
Modeling objective	Prediction? Inverse identification? Insight generation?	ANN (prediction), SVM/PCA (inverse), FEA/PINN (insight)	Prediction *R*^*2*^ should be more than 0.90	[58, 146, 150, 153]
Interpretability	Is mechanistic understanding required?	FEA, PINNs, TCPINNs, MIML	Feature importance: top features should explain more than 80% variance	[68, 179]
Computational resources	Are powerful resources available?	FE^2^, PINNs, DMNs, full µFE	Runtime 2–48 h, memory 8–128 GB RAM	[146, 152]
Biological realism	Need for patient-specific modeling or microstructural fidelity?	CNN, PCA, HFGMC, Image-informed FEM	CT resolution 2–25 µm voxels	[8, 80, 85]
Need for speed	Is real-time performance required?	ANN surrogate	Inference time 5–50 ms	[148, 152, 198]

Collectively, Table 5 provides a structured overview supporting the selection of appropriate multiscale modeling strategies based on scale, data availability, computational constraints, and desired biological realism. This framework reinforces the complementary nature of classical, data‑driven, and physics‑informed methodologies and offers a practical basis for designing integrated, hierarchical models aligned with specific research or clinical objectives.

Compared with existing reviews on multiscale bone mechanics, which have typically emphasized either classical FEM-based frameworks, this review uniquely integrates insights across classical, data driven, and hybrid physics-informed paradigms. This tri-layered perspective enables a more complete understanding of how hierarchical mechanical behavior can be simulated, predicted, and validated across scales. Additionally, by summarizing application specific strengths and limitations, and introducing a formal decision matrix (Table 5), this review provides practical guidance for selecting modeling tools based on research goals and constraints. These combined elements constitute a comprehensive and novel contribution that aims to shape future methodological development and support the transition toward clinically viable, physics grounded digital models of bone.

## Future Scope

Despite substantial progress in classical, data driven, and hybrid multiscale modeling techniques, several important scientific and technological challenges remain unresolved. Future advances in bone biomechanics will rely on the integration of higher resolution imaging, physics-informed artificial intelligence, and clinically deployable computational pipelines for patient-specific predictions. Building on the studies highlighted in this review, the following directions present the most promising opportunities for the future.

*High-fidelity imaging and microstructural data integration* advances in synchrotron phase contrast imaging, plasma focused ion beam serial sectioning, and ultra-high-resolution HR-pQCT will allow more accurate quantification of nanoscale mineral architecture, collagen orientation, and lacuna-canalicular network morphology. Incorporating these datasets into multiscale models could significantly improve predictions of crack initiation, failure propagation, and remodeling pathways. Recent developments in 3D deep learning-assisted segmentation and automated microstructure reconstruction further support this direction [199–202].

*Large scale, multimodal datasets, and open benchmarking standards* there is a pressing need for open, multiscale bone biomechanics datasets that include micro-CT, nanoindentation, DVC strain fields, and long-term clinical outcomes. Such datasets are essential for training ML surrogates and calibrating PBML frameworks. Standardized verification/validation protocols particularly for hybrid and ML-based models will be essential for clinical translation. Data fusion strategies that integrate QCT, DXA, MRI, micro-CT, and biochemical markers represent a pathway for robust model generalization across populations [142].

*Digital twins of bone and patient-specific mechanobiology* recent developments in scale bridging and dynamic analysis further extend FEM capabilities. Submodeling workflows have enabled the quantification of transient stress amplification and modal content transfer across scales [76]. These approaches represent early steps toward activity-aware digital twins of the skeletal system, which could further develop patient-specific digital twins capable of simulating whole organ mechanical behavior, adaptation, and therapeutic outcomes in real time. Such frameworks would integrate longitudinal clinical imaging, biochemical markers, and personalized mechanobiological parameters. The advances in homogenization, PBML, and computational biomechanics highlighted in this review provide a strong foundation for emerging multiscale digital twin frameworks. However, substantial challenges remain, including improved multiscale coupling, robust uncertainty quantification, and rigorous validation across heterogeneous populations [203–205].

*Scalable physics aware ML for multiscale prediction* PINNs and neural operators represent a transformative opportunity for scalable, mesh free multiscale simulations. Their ability to embed constitutive laws, remodeling kinetics, damage mechanics, and poroelasticity directly into neural architectures could drastically reduce computational burdens while maintaining physical validity. Next generation architectures such as multistage PINNs, higher order multiscale PINNs, and thermodynamics informed networks are expected to accelerate simulations of fracture healing, aging, osteoporosis progression, and implant osseointegration [170, 174]. However, improving training stability, enforcing boundary condition consistency, and integrating sparse clinical data remain active research challenges.

*Fully integrated multiphysics–multiscale frameworks* future multiscale frameworks should incorporate coupled physical fields including piezoelectricity, flexoelectricity, electromechanical signaling, and fluid–solid interactions to simulate real mechanotransduction pathways and enable prediction of implant mechanobiology, guided bone regeneration, and the influence of electrical/magnetic stimulation therapies. Early electro- and magnetomechanical bone models suggest significant translational potential, but remain underexplored [15, 73].

*Clinical integration and real time decision support systems* computational feasibility and interpretability remain major barriers to routine clinical adoption. Future work should focus on integrating FE ANN/PINN surrogates and digital twins into clinical workflows for rapid fracture risk assessment, real time implant design optimization, patient-specific rehabilitation planning, and individualized prediction of bone remodeling and degeneration. The development of lightweight inference models (e.g., surrogate ANN–FE hybrids) capable of producing millisecond scale predictions will be crucial for translation into clinical and surgical planning tools.

*Multiscale modeling of bone diseases* in addition to the mechanical and remodeling phenomena addressed in this review, the multiscale modeling frameworks discussed here also provide a strong mechanobiological and mathematical foundation for future investigations of bone diseases [206, 207]. Tissue necrosis (e.g., osteonecrosis) is closely associated with dysregulated mechanobiology, microstructural degradation, impaired remodeling, and disrupted fluid transport [208]. Methods such as ABMs [13, 25] that explicitly represent cell-level mechano-responses, including osteocyte apoptosis, altered osteoblast/osteoclast activity, and remodeling failure under perturbed mechanotransduction are well suited to capturing early cellular-level biological and mechanical dysfunctions that may precede necrosis. Similarly, VMMs and poro-micromechanical models [95, 99], which link pore-scale fluid flow to cell stimulation, offer a potential pathway to modeling the impaired lacunar-canalicular transport known to contribute to osteocyte death and necrotic tissue collapse. Furthermore, asymptotic homogenization and hierarchical micromechanics approaches [93, 96] capable of representing mineralization heterogeneity, microstructural damage evolution and anisotropic weakening could be extended to simulate the progression from micro-damage accumulation to localized necrotic lesions. Although explicit modeling of necrosis is beyond the scope of this review, these multiscale methodologies collectively offer the mechanistic tools necessary to explore its onset, progression, and mechanical consequences across hierarchical levels of bone.

## Figures and Tables

**Fig. 4:**
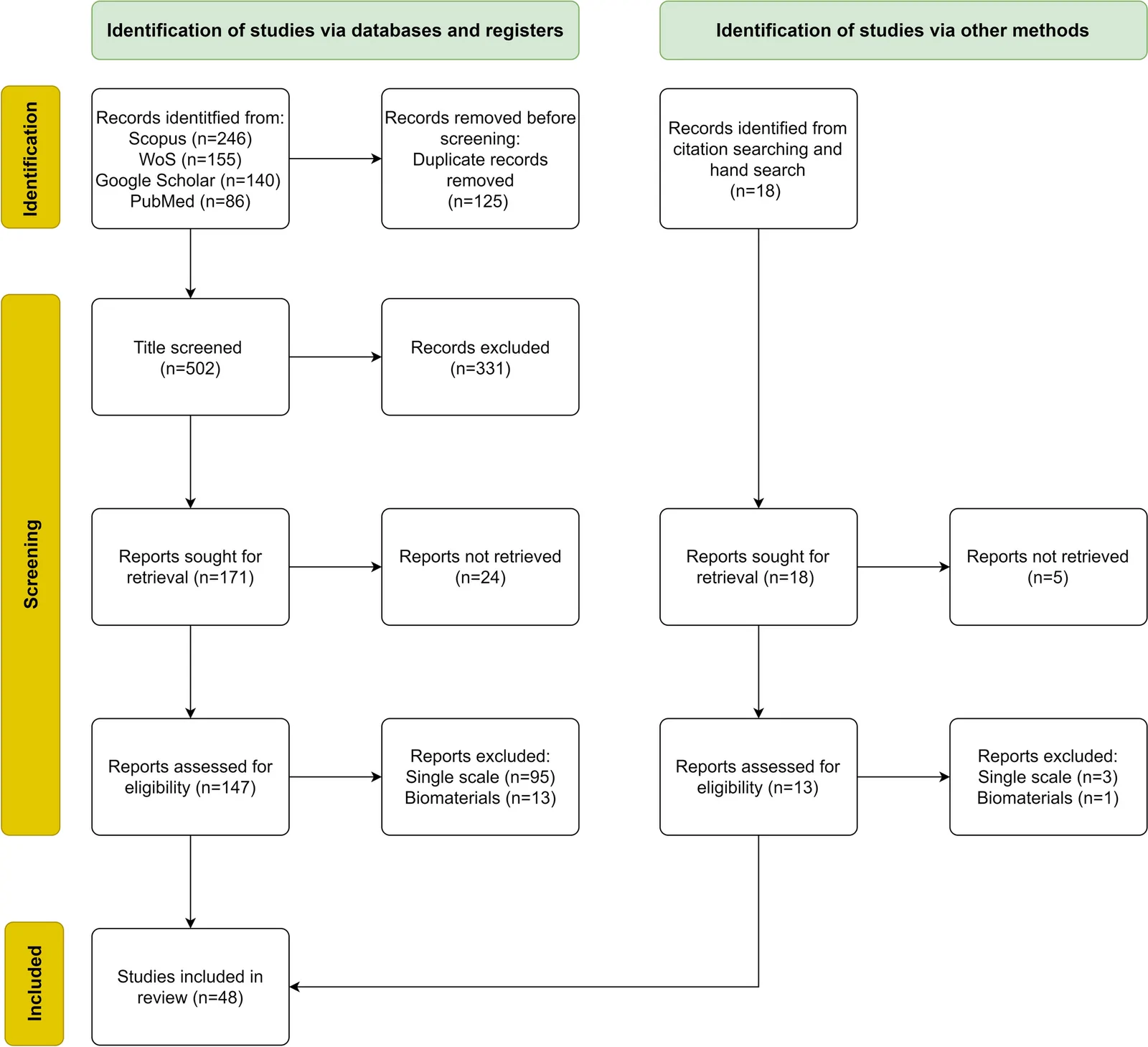
PRISMA flow diagram depicting the process of selecting studies

## Data Availability

No new data were generated or analyzed in this study. Data sharing is not applicable to this article.

## Appendix: Materials and Methods

The systematic review was based on PRISMA 2020 guidelines (Preferred Reporting Items for Systematic Reviews and Meta-Analyses) to assure transparency, reproducibility, and methodological rigor. The whole selection process, from identification to screening, assessment of eligibility, and inclusion, has been outlined in a PRISMA flow chart shown in Fig. 4.

### Literature Search Strategy

An exhaustive literature search was conducted across four major electronic databases: Scopus, Web of Science (WoS), PubMed, and Google Scholar. The search was completed in April 2025. Search strings were developed using various combinations of relevant keywords and Boolean operators, structured around three core concepts:

- Biomechanical focus: (“bone” OR “bone tissue” OR “osteon”),
- Modeling strategy: (“multiscale” OR “hierarchical”) AND (“finite element method” OR “machine learning” OR “physics-based” OR “hybrid modeling”),
- Technique type: (“data-driven” OR “classical” OR “physics-informed” OR “hybrid”).

To ensure the completeness of the review, additional manual searches were conducted, including screening the bibliographies of key articles. Keywords were required to appear in the title, abstract, or author-provided keywords of each publication.

### Inclusion and Exclusion Criteria

To be included, studies had to:

- Be published between 2015 and 2025.
- Explicitly model or simulate multiscale mechanical behavior of bone (e.g., linking nano-, micro-, and macro-scale features).
- Employ one or more of the following modeling paradigms: classical, data-driven, or hybrid.
- Provide sufficient methodological detail regarding modeling assumptions, scales, or experimental validation.

Exclusion criteria were:

- Non-English language articles.
- Studies limited to single-scale modeling without reference to scale interaction or transitions.
- Studies with insufficient information for full-text screening.
- Studies focused only on material science or biology without mechanical modeling.

### Study Selection Process

The literature selection process was conducted in accordance with PRISMA 2020 guidelines. A total of 627 records were identified across four databases: Scopus (*n* = 246), WoS (*n* = 155), Google Scholar (*n* = 140), and PubMed (*n* = 86). After excluding 125 duplicates, 502 unique records remained for title and abstract screening. Of these, 331 records were removed due to irrelevance. The remaining 171 articles were assessed for full-text eligibility. However, 24 articles could not be accessed due to various reasons, such as unpublished studies, conference abstracts or posters without full papers, and non-digitized or poorly indexed content. Of the remaining articles, 147 studies were selected for full-text screening. A total of 108 studies were excluded—95 for focusing solely on single-scale and 13 for an irrelevant focus on biomaterials. In parallel, 18 additional articles were identified through manual search and citation tracking. Of the 18 additional articles, 5 were unavailable. Among the remaining 13, 4 were excluded—3 due to a single-scale focus and 1 for addressing only biomaterials. In total, 48 studies met all inclusion criteria and were eligible for qualitative synthesis. A summary of the selection process is presented in the structured PRISMA flow diagram (Fig. 4).

### Data Categorization

From the final pool of included studies (*n* = 48), three thematic categories were identified:

- Classical techniques (*n* = 33), including 23 FEM-based models and 10 studies using alternative mechanistic approaches.
- Data-driven techniques (*n* = 13), using ML-based techniques.
- Hybrid frameworks (*n* = 2), along with an additional 16 emerging studies identified as having high potential for future multiscale applications though not yet directly applied to bone.

These categories are examined in detail throughout the review. Each study is evaluated based on key criteria, including scale bridging, computational performance, physical realism, and clinical applicability. The final dataset of 48 papers is further analyzed by the bone type (cortical and cancellous) and by mechanical modeling objectives such as damage prediction, stiffness mapping, remodeling simulation, and fracture risk estimation. This analysis highlights the broad range of spatial scales addressed by the models, from the nanoscale to the macroscale. The distribution of studies across modeling paradigms also reveals prevailing trends and emerging directions in the field, particularly the growing emphasis on combining frameworks to develop more interpretable, scalable, and clinically relevant bone models.
